# Case Report: Application of the 6D BTS digital assessment system in the comprehensive management of refractory cancer pain in a patient with gallbladder carcinoma

**DOI:** 10.3389/fpain.2026.1808441

**Published:** 2026-10-08

**Authors:** Jing Ren, Yuewen Li, Kunzhong Zhou, Yunhua Wang, Quanli Wang, Dongmei Hu, Tingting Song, Hui Dong, Zhijuan Cheng, Changrong Hu, Kunxiang Huang, Yuan Zhang, Li Zhuang

**Affiliations:** 1 The Third Affiliated Hospital of Kunming Medical University, Yunnan Cancer Hospital, Peking University Cancer Hospital Yunnan, Kunming, Yunnan, China; 2 Clinical Immunology Department, Kunming Children’s Hospital & Children’s Hospital Affiliated to Kunming Medical University, Kunming, Yunnan, China

**Keywords:** Botong score, comprehensive management, gallbladder carcinoma cancer, pain managemant, refractory cancer pain

## Abstract

Cancer pain is a major complication that adversely affects the quality of life of cancer patients, with approximately 10%–20% of patients developing refractory cancer pain that conventional medications fail to control. Traditional unidimensional assessment tools cannot fully capture the complexity of this condition. We therefore applied a novel digital tool, the 6D BTS (Botong score) digital assessment system, which was integrated with a robotic device. This system provided a multidimensional, objective evaluation through a digital interface, assessing pain intensity, physical function, emotional status, sleep quality, neuropathic pain, and breakthrough pain. The system also enabled remote dynamic monitoring outside the hospital. We report a case of gallbladder carcinoma with clavicular metastasis and recurrent sternal pain. Using a robotic system integrated with the BTS system, we conducted a comprehensive assessment of the patient's pain. Based on the detailed information provided by the BTS system, we designed a personalized analgesic plan that controlled the cancer pain to some extent: the patient's Numeric Rating Scale score decreased from 8 to 1, and breakthrough pain episodes dropped from 6 times per day to 0. We conclude that the BTS system offers an effective digital solution for the comprehensive management of refractory cancer pain.

## Introduction

Cancer pain is one of the most common and intolerable complications among cancer patients. The overall prevalence of cancer pain is 44.5%, and approximately 30.6% of patients experienced moderate to severe pain. Among patients with advanced, metastatic, or end-stage cancer, the prevalence of pain increases to 54.6% (1–3). Most patients with cancer pain achieved good analgesia through oral or topical drug administration. However, 10%–20% of those with moderate-to-severe cancer pain did not obtain effective relief. Others discontinued treatment because of intolerable side effects, which leads to pain exacerbation. These cases are defined as refractory cancer pain (4). Refractory cancer pain triggers central sensitization, placing the nervous system in a state of abnormally heightened and sustained sensitivity to pain. Within this vicious cycle, pain not only causes direct suffering but also amplifies pain signals through emotional and physiological mechanisms, leading to continuous deterioration of the condition (5). Thus, accurate and comprehensive assessment of cancer pain is essential for its diagnosis and treatment (6).

Botong score (BTS), a digital multidimensional pain assessment tool, comprised six dimensions: NRS pain score; pain's impact on physical function, mood, and sleep; assessment of neuropathic pain; and assessment of breakthrough pain. The NRS score ranged from 0 to 10. A score of 0 indicated no pain. Scores of 1–3 reflected mild pain, 4–6 moderate pain, and 7–10 severe pain. The impact scores for physical function, mood, and sleep also ranged from 0 to 10. A score of 0 signified no impact, whereas a score of 10 signified an extremely large impact. We assessed neuropathic pain using the LANSS scale (7). Affirmative responses to questions 1 through 5 each added one point; an affirmative response to question 6 subtracted one point. A total score of 0 ruled out neuropathic pain. A score of 1 did not fully exclude it. Scores of 2–3 suggested neuropathic pain, and scores of 4–5 strongly suggested neuropathic pain. Table 1 presents the detailed scoring criteria (8). Reliability analysis yielded a Cronbach's *α* coefficient of 0.78 for the BTS, and validity analysis gave a KMO value of 0.80 (9). These findings demonstrate good reliability and validity. A robot equipped with the BTS 6D system visited each patient's bedside, administered the questionnaire, and organized the results. Based on the patient's scores and current clinical guidelines, the robot provided tailored recommendations. The robot engaged patients effectively, remained unaffected by healthcare staff, and delivered standardized, objective scoring with a high detection rate.

**Table 1:** Content of the multi-dimension Botong score.

Questions	Options	Notes
1. Numeric rating scale (NRS): Please evaluate your pain score	0–10	0 (no pain), 10 (the worst pain imaginable)
2. Impact of pain on function		
2.1 Please evaluate the impact of pain on your cervical vertebra function	0–10	0 (no impact), 10 (the most serious impact)
2.2 Please evaluate the impact of pain on your upper limb function	0–10	0 (no impact), 10 (the most serious impact)
2.3 Please evaluate the impact of pain on your lower back function	0–10	0 (no impact), 10 (the most serious impact)
2.4 Please evaluate the impact of pain on your lower limb function	0–10	0 (no impact), 10 (the most serious impact)
3. Please evaluate the impact of pain on your emotions	0–10	0 (no impact), 10 (the most serious impact)
4. Please evaluate the impact of pain on your sleep	0–10	0 (no impact), 10 (the most serious impact)
5. Do you have the following symptoms of pain		
5.1 Needling stabbing pain	Yes or no	
5.2 Burning or flaming pain	Yes or no	
5.3 Pain with a tingling sensation	Yes or no	
5.4 Pain feels like an electric shock	Yes or no	
5.5 Pain exacerbated by touching clothes or sheets	Yes or no	
5.6 Pain only occurs in the joints	Yes or no	
6. Breakthrough pain assessment: If you are already being treated with analgesics and the pain is under control, have you experienced sudden, brief, or unbearable severe pain (including when your body position changes)?		

In this case report, we describe a patient with gallbladder cancer that metastasized to the clavicle. The patient experienced recurrent sternum pain. After admission, we evaluated the patient using the BTS 6D system. This assessment significantly controlled the patient's cancer pain. Through this case, we observed that this digital tool demonstrated significant potential in improving the accuracy of pain assessment, optimizing analgesic strategies, and enhancing the overall quality of patient care, providing a new practical approach for the precise management of clinically refractory cancer pain.

## Case description

A 70-year-old male underwent laparoscopic cholecystectomy with extended radical resection for gallbladder carcinoma and partial hepatectomy in February 2022 at another hospital. Histopathological examination revealed moderately to well-differentiated adenocarcinoma of the gallbladder, with invasion of the serosal layer, perineural invasion, no vascular invasion, and carcinoma involvement of the gallbladder neck margin. Two out of two lymph nodes at the porta hepatis showed metastases. The postoperative pathological stage was determined as T3N1Mx. Postoperatively, the patient received four cycles of oral capecitabine chemotherapy, administered intermittently. In October 2022, clavicular metastasis was detected, and the patient underwent 25 sessions of localized radiotherapy at the referring hospital. Following radiotherapy, one additional cycle of chemotherapy (specific regimen unknown) was given. However, chemotherapy was discontinued due to significant thrombocytopenia. In May 2023, the patient presented with chest pain. Imaging studies at the referring hospital suggested sternal metastasis. Subsequently, the patient underwent 25 sessions of localized radiotherapy targeting the sternum and received two cycles of chemotherapy with a GP regimen during the radiotherapy period. The final chemotherapy session occurred on August 16, 2023. Severe myelosuppression was observed after each cycle, leading to the discontinuation of further chemotherapy.

Over the past year, the patient experienced intermittent stabbing chest pain, initially self-managing symptoms with compound codeine phosphate and ibuprofen sustained release tablets (2 tablets bid). As the pain intensified, the patient sought care at an external hospital, where the treatment regimen was escalated to oxycodone hydrochloride extended-release tablets (10 mg, q12h). With further pain progression, the regimen was adjusted to oxycodone hydrochloride extended-release tablets (50 mg, q12h) in combination with pregabalin (75 mg, qd) and celecoxib (200 mg, q12h). Morphine sulfate suppositories (20 mg per rectum) were intermittently prescribed for breakthrough pain. In the five days prior to presentation at our department, the patient reported a marked increase in pain intensity, which significantly impaired sleep and appetite, prompting his initial evaluation and treatment in our facility. On admission, the patient appeared cachectic with poor overall condition and mental state. He was alert but physically weak, with a Karnofsky Performance Status (KPS) score of 60. Vital signs showed a heart rate of 90 beats per minute with regular rhythm, and there was no evidence of water-hammer pulse, pulsus paradoxus, or capillary pulsation. The patient maintained a forced supine position. Chest examination revealed post-radiation hyperpigmentation and two palpable subcutaneous nodules, each approximately 2 cm in diameter and firm in texture. The right supraclavicular fossa revealed an enlarged lymph node measuring approximately 1.5 cm, firm and tender to palpation. Lung auscultation demonstrated clear breath sounds bilaterally, though fine crackles were noted in the right lung fields. Examination of the lower extremities revealed pitting edema. The primary diagnoses on admission were: (1) gallbladder carcinoma (rpT3N2M1, Stage IVB); (2) chronic, refractory chest and back pain (pain score: 8/10); (3) multiple secondary bone malignancies; (4) secondary hepatic malignancies; (5) secondary pulmonary malignancies; and (6) secondary left adrenal malignancy.

Upon admission, we performed a comprehensive bedside pain assessment using a robotic-assisted system (BTS). The evaluation identified the patient's primary pain as localized to the chest and back, characterized by stabbing and burning sensations with an overall Numeric Rating Scale (NRS) score of 8/10. Specific regional pain scores were 4 for the cervical spine, 3 for the upper limbs, 6 for the lumbar and dorsal regions, and 7 for the lower limbs. The pain profoundly impacted the patient's sleep (score 9) and emotional well-being (score 10). Screening identified a neuropathic pain component (score 3), and the patient experienced six episodes of breakthrough pain daily. Furthermore, the patient exhibited incident pain significantly exacerbated by postural changes. These high pain scores and their substantial interference with daily functioning underscored the refractory nature of the condition. In accordance with the *2017 Expert Consensus on Refractory Cancer Pain* (10), we developed a tailored, multimodal analgesic regimen to address the patient's complex pain profile and functional impairment.

On January 3, 2024, we initiated intravenous hydromorphone patient-controlled analgesia (PCA) using a concentration of 0.6 mg/mL (120 mg in 200 mL normal saline). The initial settings included a 1.3 mL/h basal rate, a 3.2 mL bolus, a 15-minute lockout interval, and an hourly limit of 10.9 mL. While baseline pain control was initially adequate, the patient experienced three breakthrough pain (BTP) episodes within the first 12 h. On January 5, we titrated the regimen to a 1.5 mL/h basal rate and a 3.6 mL bolus (12.3 mL hourly limit), resulting in a NRS score of 3 and three daily BTP episodes. By January 7, the patient reported increased pain (NRS 6) with six daily BTP episodes, occurring predominantly at night. We subsequently added pregabalin and olanzapine and adjusted the PCA to a 2.5 mL/h basal rate and a 4.5 mL bolus (16 mL hourly limit). This achieved stable pain control (NRS 3) with only one daily BTP episode. We identified the pain as classic incident pain triggered by movement or postural changes; therefore, we instructed the patient to self-administer pre-emptive PCA boluses before physical activity. Finally, we prescribed laxatives and dietary modifications to prevent opioid-induced constipation.

On January 13, 2024, the patient transferred to a local hospital while continuing intravenous PCA. At the time of discharge, the patient's NRS score was 1, and no breakthrough pain episodes had occurred. We monitored his progress post-discharge using the BTS digital health system. By January 20, 2024, the patient maintained stable analgesia with an NRS score of 2. Compared with admission, impact scores for regional pain and daily functioning decreased significantly: cervical spine (score 2), upper limbs (score 0), lumbar-dorsal region (score 1), lower limbs (score 1), sleep (score 0), and emotional state (score 1). Neuropathic pain decreased to a score of 1, and breakthrough pain remained absent. This standardized analgesic management successfully improved the patient's sleep and mood, leading to a marked enhancement in his overall quality of life (Figures 1, 2).

**Figure 1:**
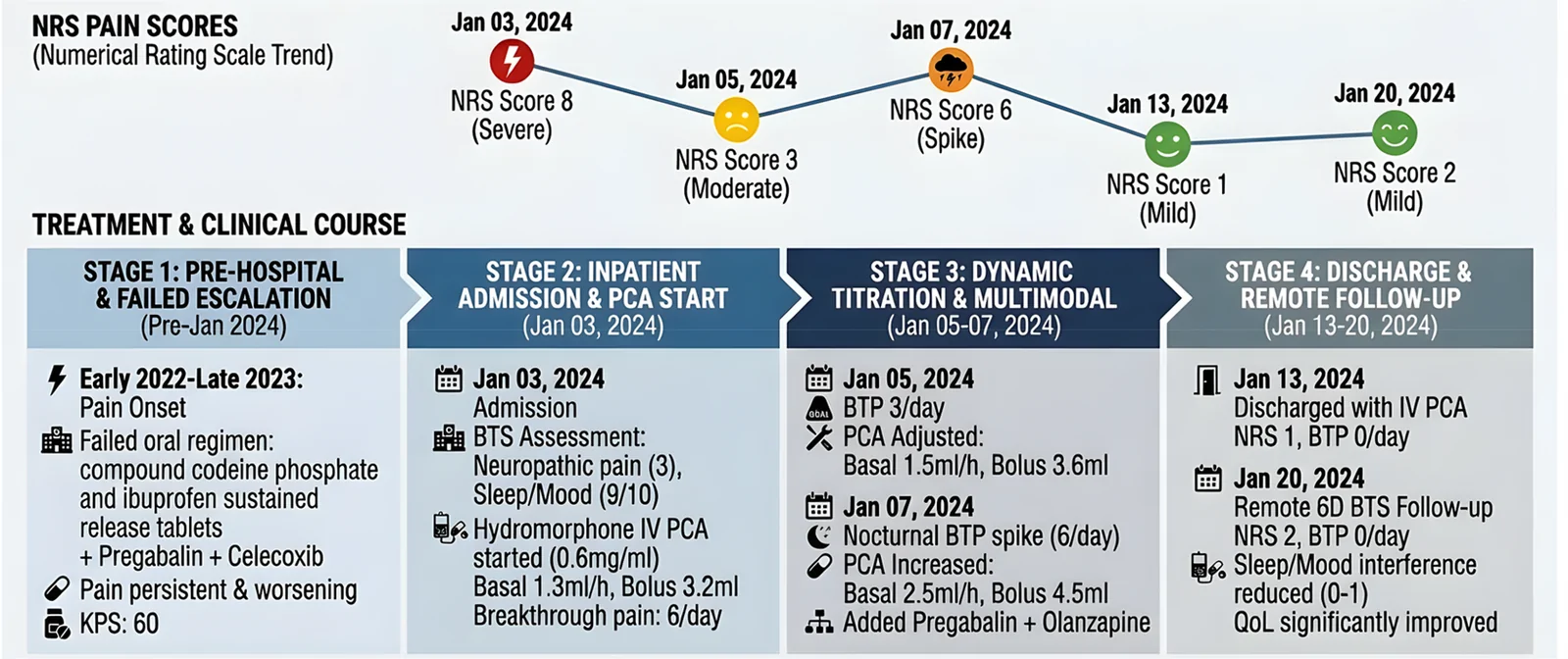
A clinical timeline figure showing the disease course, prior treatments, and NRS assessment time points.

**Figure 2:**
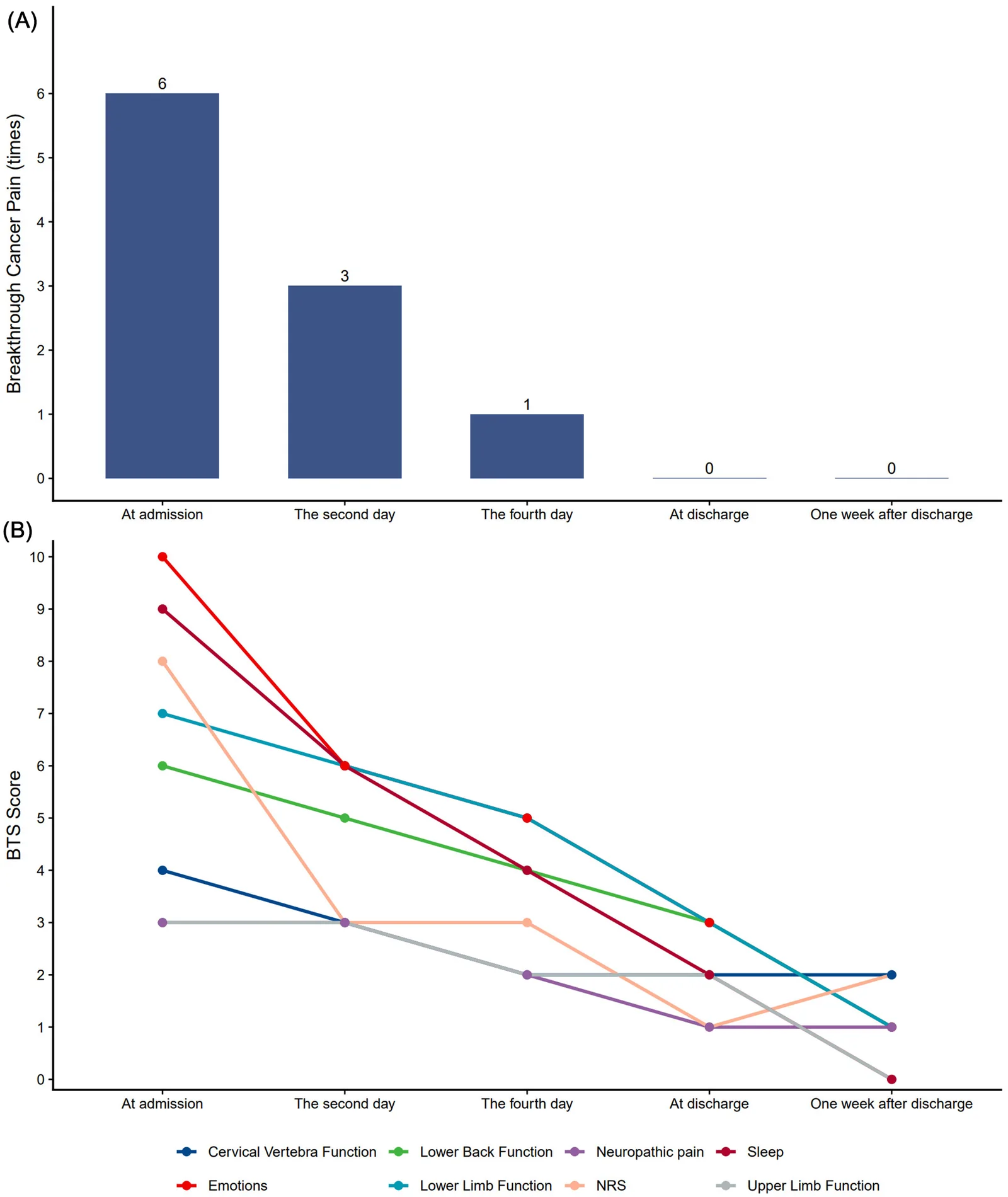
**(A)** The frequency of breakthrough cancer pain. **(B)** Showing BTS across all domains at each assessment point (At admission, the second day, the fourth day, At discharge, One week after discharge).

## Discussion

Cancer pain remained a prevalent and debilitating symptom of malignancy, arising from primary tumor invasion, metastatic disease, treatment-induced nerve injury, and psychological distress. Although clinicians widely utilized the World Health Organization (WHO) analgesic ladder (11), 10%–20% of patients progressed to refractory cancer pain (12). Persistent, severe pain triggered detrimental systemic responses—including elevated stress hormones, increased cardiovascular demand, and impaired immune function—which potentially accelerated disease progression (13). Consequently, multidimensional monitoring throughout the disease course was essential to improve quality of life and preserve patient dignity. In this report, we detailed the application of the digital “6D BTS” assessment system in managing a patient with advanced gallbladder cancer and refractory pain. We observed that this digital tool significantly improved assessment precision, optimized analgesic strategies, and enhanced the overall quality of care. This case might offer some references for the precise management of intractable cancer pain.

Current clinical research relied predominantly on subjective assessment methods. These included unidimensional tools, such as the NRS (14)and Visual Analogue Scale (VAS) (15), as well as multidimensional instruments like the BPI and neuropathic pain screening scales. However, these tools often exhibited limited reliability and validity. Because patient subjectivity inherently biased these scores, resulting treatment regimens frequently deviated from optimal protocols. While neurophysiological testing offered objective data, high equipment costs and technical complexity limited its widespread clinical utility. Consequently, an urgent need arose for a digital assessment tool that integrated multidimensional depth with objectivity and clinical ease of use in cancer pain management.

In this case, we utilized the 6D BTS system to expand pain assessment beyond simple intensity into six critical dimensions: physical function, emotional state, sleep quality, neuropathic components, and breakthrough pain frequency. This comprehensive approach enabled us to identify nerve-compression-related neuropathic pain, severe sleep deprivation, and secondary anxiety. In addition, the BTS system may be able to help make up for some of the deficiencies of the BPI; while the BPI requires complex color-coding on body maps—a task frequently too difficult for elderly patients—the BTS system employed voice-activated control and simplified queries to ensure accessibility. By integrating artificial intelligence with evidence-based guidelines, the system can assist in providing a diagnostic framework, which is helpful for formulating highly personalized analgesic regimens.

Through the robot interface, the BTS 6D system conducted a comprehensive assessment of the patient's cancer pain condition. For this patient with gallbladder cancer, the BTS identified a resting pain score of 8/10 and critical neuropathic features related to nerve compression. The system functioned as a diagnostic alert for severe sleep deprivation and secondary anxiety, which transitioned our therapeutic goals from isolated analgesia to integrated, humanistic care. Furthermore, the system's predictive analytics indicated that opioid monotherapy would be insufficient, providing a quantifiable rationale for initiating adjunctive gabapentinoids. By integrating artificial intelligence with evidence-based guidelines, the BTS system may offer some assistance in formulating personalized pain relief strategies.

While patient-controlled analgesia (PCA) offered significant benefits for refractory cancer pain, conventional monitoring lacked continuous, full-cycle feedback. Patients often struggled to self-assess demand-dosing requirements, particularly for incident pain, where professional guidance was frequently delayed (16). To address this therapeutic gap, we utilized the robotic BTS system for comprehensive, dynamic assessments. This system established a foundation for precision pain management and simplified post-discharge follow-up. Furthermore, the robotic interface reduced the clinical workload for healthcare staff. The integration of digital assessment with hydromorphone PCA facilitated rapid, effective analgesia. This individualized, data-driven approach reduced the risk of dosing errors and adverse events, ultimately enhancing the patient's quality of life and alleviating anxiety.

## Conclusions

To our knowledge, this represented the first clinical application of the 6D BTS system to guide analgesic management in a patient with refractory gallbladder cancer pain. The intervention has improved the patient's pain control. However, the patient succumbed to disease progression one month after discharge, which precluded the collection of long-term follow-up data. Despite this limitation, the case demonstrated that the BTS system's multidimensional evaluation—encompassing pain intensity, physical function, emotion, sleep, neuropathic components, and breakthrough pain—enhanced clinical efficiency through its integrated analytical reports. In this case, multidimensional digital assessment proved feasible and provided clinically relevant information, which may offer a foundation for hypothesis testing in subsequent controlled trials. Going forward, investigations should focus on full-cycle management and real-time surveillance of refractory pain, thereby maximizing the quality of life of patients with cancer.

## Patient’s perspective

Before treatment, the patient reported that his pain “destroyed my will to live.” He specifically stated that his greatest fear was not death, but “dying in agony.” After achieving adequate analgesia, he told the nursing staff, “Now I can say goodbye to my children without screaming in pain.” This patient-reported experience highlights that the real harm of cancer pain lies not only in physical suffering but also in the erosion of dignity and meaningful connection at the end of life.

## Claims and limitations

As the BTS is an assessment instrument, the observed analgesic benefit is attributable to the therapeutic regimen it informed rather than to the tool itself; this single case cannot isolate the incremental contribution of multidimensional assessment over standard titration.

## Data Availability

The raw data supporting the conclusions of this article will be made available by the authors, without undue reservation.
